# Prevalence, factors and quality of life associated with frailty and pre‐frailty in middle‐aged and older adults living with HIV in Zimbabwe: A cross‐sectional study

**DOI:** 10.1111/hiv.13716

**Published:** 2024-09-20

**Authors:** Anthony Muchai Manyara, Tadios Manyanga, Anya Burton, Hannah Wilson, Joseph Chipanga, Tsitsi Bandason, Chris Grundy, Etheldreda I. Yoliswa Madela, Kate A. Ward, Bilkish Cassim, Rashida Abbas Ferrand, Celia L. Gregson

**Affiliations:** ^1^ Global Health and Ageing Research Unit, Bristol Medical School University of Bristol Bristol UK; ^2^ The Health Research Unit Zimbabwe Biomedical Research and Training Institute Harare Zimbabwe; ^3^ MRC International Statistics and Epidemiology Group, London School of Hygiene and Tropical Medicine London UK; ^4^ Department of Geriatrics University of KwaZulu‐Natal Durban South Africa; ^5^ MRC Unit The Gambia, London School of Hygiene and Tropical Medicine Banjul The Gambia; ^6^ MRC Life course Epidemiology Centre University of Southampton Southampton UK; ^7^ Clinical Research Department, London School of Hygiene and Tropical Medicine London UK

**Keywords:** Africa, ageing, frailty, HIV, HRQoL, Zimbabwe

## Abstract

**Objectives:**

We investigated associations between HIV, frailty and health‐related quality of life (HRQoL).

**Methods:**

This cross‐sectional study recruited men and women aged ≥40 years in Zimbabwe. A researcher collected clinical and HRQoL data, and performed physical assessments and HIV testing. Frailty was defined using five criteria: unintentional weight loss, exhaustion, low physical activity, low gait speed, low handgrip strength. The presence of three or more criteria defined frailty, one to two pre‐frailty, and zero non‐frail. Data analysis used adjusted regression modelling.

**Results:**

Of 1034 adults (mean ± SD, 62.0 ± 14.0 years), 21.6% (*n* = 223) were living with HIV: 93.3% knew their status, of whom 96.2% were on antiretroviral therapy (ART) and 89.7% of these had a viral load <50 copies/mL. Mean age at HIV diagnosis was 44.6 ± 10.4 years (only 8.1% were ≥70 years), people had been living with HIV for 9.8 ± 5.0 years and had been on ART for 9.4 ± 5.2 years. Overall, HIV was not associated with frailty: adjusted odds ratio (aOR) was 0.99 [95% confidence interval (CI): 0.42–2.33] for frailty versus non‐frailty. However, each 5 years lived with HIV was associated with twice the odds of frailty/pre‐frailty (aOR = 2.03, 95% CI: 1.03–4.13), independent of age and ART duration. Furthermore, each 5 years of ART use was associated with 60% lower odds of frailty/pre‐frailty (aOR = 0.39, 95% CI: 0.19–0.78), independent of age and years lived with HIV. Older age, minimal education and poverty were associated with frailty. Frailty was associated with lower HRQoL in people both with and without HIV.

**Conclusion:**

Reduced survival and good viral suppression may explain the lack of association between HIV and frailty. Early ART initiation could reduce future risk of frailty.

## INTRODUCTION

The older African population is growing rapidly against a backdrop of better infectious disease control and improved nutrition, but increasing age‐related noncommunicable diseases [1]. Antiretroviral therapy (ART) roll‐out has made an important contribution to these demographic shifts [1, 2] such that many more people are ageing with HIV. Aside from HIV, Africa's older population faces multiple health challenges compounded by limited access to healthcare [1]. Physical frailty (frailty hereafter) is a clinical syndrome characterized by weakness, reduced endurance and physical function, which increase disability, morbidity and ultimately mortality [3, 4]. Pre‐frailty is an intermediate stage between frailty and non‐frailty associated with progression to frailty and adverse health outcomes, albeit to a lesser extent compared with frailty [3]. Both frailty and pre‐frailty can be prevented and/or treated, e.g. through dietary and exercise interventions [4].

A recent meta‐analysis of 240 studies from 62 countries (only six in Africa included) in people aged ≥50 years found pooled prevalence rates of frailty and pre‐frailty of 12% and 46%, respectively, with frailty being more common in the African region (22%) but pre‐frailty being similar to the global prevalence (47%) [5]. A potential contributor to a high prevalence of frailty in Africa is the high HIV prevalence [6]. Living with HIV increases the risk of chronic inflammation, comorbid disease and exposure to psychosocial and environmental stressors (e.g. malnutrition, substance abuse), which can contribute to frailty [7, 8]. However, the complexity and multiplicity of contributors to frailty mean that underlying mechanisms linking HIV infection, ART and frailty remain unclear [8].

Evidence concerning the association between HIV and frailty in Africa is scant. A systematic review in 2017 on frailty in people living with HIV (PLWH) found only two relevant, but conflicting, case–control studies in sub‐Saharan Africa [9]. In Senegal, HIV infection was not associated with frailty: frailty was lower in PLWH established on ART for ~10 years (7/202; 3.5%) than in people without HIV (14/202, 6.9%) [10]. In contrast, in South Africa, PLWH on ART for a median of 5 years were more likely to be frail than those without HIV; notably a CD4 count <500 cells/μL was associated with increased frailty risk [11]. The few studies since 2017 have reported mixed results. In 2020, a South African case–control study found no association between HIV and frailty [12]. The following year, a cross‐sectional study in rural Tanzania recruiting adults with HIV aged ≥50 years (72.6% virally suppressed) reported a 3% prevalence of frailty which rose to 7% prevalence in those aged ≥60 years [13]; this was lower than previously reported in community‐dwelling adults aged ≥60 years in cross‐sectional studies in rural Tanzania (9% in one study and 19% in another) [14, 15]. In 2023, an Ethiopian matched case–control study found those living with HIV (for a median of 13 years) were more frail than those without HIV [16]. Therefore, the association between HIV and frailty in Africa remains unclear, and many populations remain unstudied [8].

In 2017, the Zimbabwean Ministry of Health and Childcare acknowledged the growing older adult population and committed to promoting healthy ageing [17]. Successful ART programmes have improved longevity: according to the Joint United Nations Programme on HIV/AIDS (UNAIDS), Zimbabwe is one of five countries in sub‐Saharan Africa to have achieved the 95–95–95 targets [2]. Currently, HIV prevalence in adults is estimated as 12.9% [18], but is higher in older adults: ≥20% in those aged 40–64 years, 13.6% in those 65–69, and 8.1% in those 70–74 years [19]. It remains to be determined whether HIV is associated with frailty, whether ART treatment and viral suppression reduce the risk of frailty in PLWH, and ultimately, whether frailty is associated with health‐related quality of life (HRQoL). Therefore, this study aimed to assess the prevalence of frailty in older adults living with and without HIV, and in PLWH, to determine the factors associated with frailty, and the association between frailty and HRQoL.

## METHODS

### Study design and population

This study is reported according to the STROBE (Strengthening the reporting of observational studies in epidemiology) checklist for cross‐sectional studies [20] (see checklist in the Data S1). It formed part of the Fractures‐E3 (Fractures in sub‐Saharan Africa: epidemiology, economic impact, and ethnography) study conducted in three countries in Africa, including Zimbabwe [21]. The current study was a cross‐sectional population‐based survey conducted between February and November 2022 in Harare, aiming to recruit 168 women and 168 men in each of three age strata (40–54, 55–69 and ≥70 years), resulting in a total of 504 women and 504 men. A sample of 168 women and 168 men provided 90% power to detect an odds ratio of 2.0 for an association between a risk factor with 13% prevalence (e.g. HIV) and an outcome seen in 9% (e.g. spine fracture) [21].

Geographic Information Systems were used to map three peri‐urban high‐density communities (Dziwarasekwa, Mufakose, Highfields). The sample size within each community was weighted according to the number of adult men aged over 70 years (from census data), as they were the least populous. ‘Blocks’ of households large enough to recruit 30 eligible individuals (5 per strata) were defined and randomly selected. Each household was sequentially enumerated, and eligible individuals invited to participate, until each age‐sex strata in the block was complete. If a potential participant was cognitively impaired, a proxy was invited to consent on their behalf. In total, 1836 households were approached, 2304 people were identified aged ≥40 years, of whom 1619 were invited to participate, with 1109 (68.5%) of those accepting the invitation, attending the research clinic, and providing consent/assent to take part in the study. They are included in the analysis (see Supplementary Figure 1).

### Data collection procedures and definitions

Data were collected using validated and standardized procedures via researcher‐administered questionnaires [sociodemographic characteristics, HIV history, comorbidities, mental health, physical performance, physical activity, lifestyle (diet, smoking, alcohol intake)]. Physical assessments (e.g. handgrip strength, gait speed, anthropometry) and blood tests (for HIV, blood glucose) were performed (see Data S1 and published protocol for more details on data collection procedures [21]).

#### Frailty definition

Frailty was determined using the five criteria proposed by Fried et al [3]: self‐reported unintentional weight loss, self‐reported exhaustion, self‐reported low physical activity, low gait speed and low grip strength. Table 1 shows the five criteria as proposed by Fried et al. when applied to the Cardiovascular Health Study in the US [3], with the adaptations made for the Zimbabwean study population. The presence of any three criteria defined frailty, one or two criteria defined pre‐frailty, and the absence of any criteria defined non‐frailty (known as robust) [3]. As per Fried et al., participants with data available for three or more out of five criteria (*n* = 1108 [99.9%]) were included in the analysis.

**TABLE 1:** Criteria and operational definition of frailty.

Criteria	Operationalization of the frailty criteria		
	Cardiovascular Health Study [3]	Zimbabwe peri‐urban population	Justification
Weight loss	Self‐reported loss of >10 lb (4.55 kg) unintentionally in the last year or ≥5% measured weight loss when compared with previous year's body weight	Self‐report to ‘Have you, or those close to you, noticed that you have lost weight or become thinner in the last 12 months?’	Self‐measurement of weight is uncommon, access to scales very limited, no routine data are collected to provide a baseline weight in this setting. Hence, we took a pragmatic approach of ‘noticeable weight loss’ by participant or someone close to them.
Exhaustion	Self‐reported based on two questions from the CES–D Depression Scale, the following two statements: In the last week: (a) I felt that everything I did was an effort; (b) I could not get going.	Self‐reported based on one question of the Shona Symptom Questionnaire [22], which translates to mean, ‘I felt run down’.	This question was included in the validated Shona Symptom Questionnaire [22] used to measure common mental disorders in this setting. It aims to identify fatigue, which can accompany depression.
Low physical activity	Self‐reported based on the Minnesota Leisure Time Activity Questionnaire. Kilocalories (kcal) of activity per week estimated, and the lowest 20% identified as meeting the frailty criteria.	Self‐reported based on the International Physical Activity Questionnaire‐Short Form [23]. Metabolic equivalent (METS) min/week calculated [24] and converted to kcal/week. Cut‐offs propose by Fried et al. [3] used: men using <383 kcal/week and women using <270 kcal/week	The International Physical Activity Questionnaire‐Short Form has been used in Zimbabwe previously [25] and is more contextually appropriate than the Minnesota Leisure Time Activity Questionnaire.
Low gait speed	Measured time to walk 15 ft (4.57 m), slowest 20% by sex and median height identified as meeting the frailty criteria.	Mean measured time of two 4‐m (13.1 ft) walks, slowest 20% by sex and median height: ***Men*** Height ≤171.1 cm: ≥6.3 s Height >171.1 cm: ≥5.6 s ***Women*** Height ≤159.7 cm: ≥8.3 s Height >159.7 cm: ≥6.6 s Further, those who did not complete the walk test and (i) self‐reported immobility or mobility only with aids *and* (ii) moderate to extreme problems with mobility *and* (iii) 0 walking MET‐min/week, were considered to meet the frailty criteria.	The Fried et al. [3] criteria (i.e. slowest 20%) was applied to this population considering the sex‐specific median height. In those missing gait speed (with reason being declined to be measured), we classified gait speed as low in those reporting mobility challenges^a^ and zero walking METS.
Low grip strength	Maximal measured grip strength, stratified by sex and BMI: lowest 20% identified as meeting the frailty criteria.	Maximal grip strength of six measures was used. Low grip strength defined as ≥2 SDs below the mean grip strength [26] of the 40–44 year age group in men and women: i.e. for men, ≤31.4 kg; for women, ≤23.7 kg Those who declined the grip strength test because they felt too weak, unsafe or unable, had shaky hands or a stroke limiting use were classified as having low grip strength.	Overweight and obesity were common. Grip strength was more strongly correlated with age (correlation coefficient = 0.49) than BMI (correlation coefficient = 0.09) in this population hence use of this criteria rather than use of BMI strata as was used in the Cardiovascular Health Study.

Abbreviations: BMI, body mass index; CES‐D, Centre for Epidemiologic Studies Depression Scale; SD, standard deviation.

^a^ Use of walking aids (sticks, crutches, frame) or immobile or reporting moderate to severe problems in walking about.

#### HIV diagnosis

Participants who had never had an HIV test or had previously tested negative were offered an HIV test. HIV infection status was based on a self‐report of having tested positive previously, or newly diagnosed HIV in the research clinic [21]. Positive diagnosis was based on confirmation by two different rapid point‐of‐care tests (Alere Determine® HIV‐1/2 from Abott Laboratories and Chembio® from Chembio Diagnostics, New York, Illinois) [21]. HIV viral load was measured for all PLWH, with viral suppression defined as <50 copies/mL [27]. Duration since HIV diagnosis and of ART were based on clinical records or self‐report.

#### Comorbid diseases and lifestyle factors

Other diseases were considered present if participants: (1) self‐reported a previous diagnosis by a healthworker, (2) self‐reported medication use for a disease, or (3) were diagnosed based on a study measurement (random blood glucose and blood pressure measurement for diabetes and hypertension, respectively). See Data S1 for definitions and data collection procedures related to all diseases. To describe comorbid disease, groups were defined as follows: neurodegenerative (stroke, dementia, epilepsy, amnesia); cardiometabolic and renal [hypertension, hypercholesterolaemia, cardiovascular, diabetes, kidney disease, cardiac disease (‘heart problem’), atrial fibrillation]; joint diseases (arthritis, gout); respiratory disease (asthma, emphysema or ‘breathing problems’); cancer (any site); history of tuberculosis; and mental health disorders (score of ≥8 on the Shona Symptom Questionnaire [22] or a self‐reported diagnosis of depression, anxiety or schizophrenia, or medication to treat a mental health disorder). Sensory loss was defined as having either visual or hearing impairment (see Data S1 for definitions). Tobacco use was self‐reported as ‘current’, ‘former’ or ‘never’. Current alcohol consumption was classified as ‘yes’ or ‘no’ based on response to the question ‘Do you drink alcoholic beverages?’

Body mass index (BMI) was calculated based on participants' height and weight and categorized as underweight (<18.5 kg m^2^), normal (18.5–24.9 kg m^2^), overweight (>25 and <30 kg m^2^) and obese (≥30 kg m^2^).

#### Wealth index

A wealth index was generated using a principal component analysis [28, 29] (see Data S1 for detailed description). Quantifying wealth using this approach has been established as a valid and robust measure of wealth in Zimbabwe [30].

#### Health‐related quality of life

Health‐related quality of life was assessed using the five questions of the EuroQol‐5 Dimension 5 Level **(**EQ‐5D‐5L) [31] questionnaire and computed to a single index value, using the ‘eq5d’ package in R [32], based on the Zimbabwe value set [33].

### Statistical analysis

Analyses were performed using R (version 4.3.1) [34]. Descriptive data are presented as mean ± standard deviation for continuous variables, and counts (and percentages) for categorical variables. Differences in descriptive data between people with and without HIV were assessed using Mantel–Haenszel tests for categorical variables or analysis of variance (ANOVA) for continuous variables, adjusted for age‐group given that PLWH were younger than those without HIV. The prevalence [and 95% confidence intervals (CIs)] of the five frailty criteria indicators and combined pre‐frailty and frailty status were assessed using estimated marginal means based on logistic regression models with an HIV status interaction term. Dependent upon proportional odds assumption tests, either multinomial or ordinal logistic regression models were used to assess the associations between HIV and frailty in the entire sample, and then factors associated with frailty in PLWH; results for both were presented as odds ratios (ORs) and 95% CIs.

A direct acyclic graph (DAG), informed by the literature and authors' background knowledge, was constructed using the DAGitty tool [23] and used to identify potential confounders of the association between HIV and frailty (see Figure S1). Aside from age, potential confounders included sex, education level, marital status, income level, malnutrition, obesity, neurodegenerative morbidity, tuberculosis, cancer and cardiometabolic morbidity, and HIV viral suppression. Potential confounders were adjusted for if they wer associated with both frailty and HIV at a *p*‐value of <0.2. The association between HIV and frailty was assessed using three models: the first was unadjusted, the second was adjusted *a priori* for age (as a continuous variable), and the third was adjusted for age and additional potential confounders (i.e. education level, marital status, BMI, wealth index and diagnosed cancer). The factors associated with frailty among people with HIV were assessed individually – in the first model unadjusted, and in the second, adjusted for age (as a continuous variable). When assessing the association between frailty and years and proportion of life since diagnosis, further adjustment was made for ART duration, and when assessing the association between frailty and ART duration, further adjustment was made for years since diagnosis – the ORs for both associations were scaled for 5‐year periods. The association between frailty and HRQoL, with an HIV status interaction, was assessed using linear regression.

## RESULTS

### Study population characteristics

Figure S1 shows the participant flow chart. Of the 93.2% (1034/1109) with an available HIV status, 21.6% (*n* = 223) were living with HIV; of these, 93.3% (208/223) knew their status and 6.7% (15/223) were newly diagnosed by the research team. These 15 individuals ranged in age from 40 to 83 years (mean ± SD 55.5 ± 11.3). Of those with known HIV, 96.2% (200/208) were established on ART, and 89.7% (175/195) of those on ART had a viral load <50 copies/mL. On average, those with known HIV had been diagnosed at age 44.6 ± 10.4 years, had lived with HIV for 9.8 ± 5.0 years, and had been on ART for 9.4 ± 5.2 years. People living with HIV were, on average, a decade younger than those without HIV (54.2 ± 9.5 vs. 64.1 ± 14.3 years, *p* < 0.001), and hence just 8.1% of those with HIV were aged ≥70 years, compared with 40.6% of those without HIV (Table 2).

**TABLE 2:** Characteristics of participants living with and without HIV, disaggregated by the three age strata.

	People living with HIV (*n* = 223, 21.6%)				People without HIV (*n* = 811, 78.4%)			
	*N* = 223	40–54 (*n* = 115, 51.6%)	55–69 (*n* = 90, 40.4%)	70+ (*n* = 18, 8.1%)	*N* = 811	40–54 (*n* = 235, 29.0%)	55–69 (*n* = 247, 30.5%)	70+ (*n* = 329, 40.6%)
**Sociodemographic characteristics**								
**Female sex**	116 (52.0%)	63 (54.8%)	43 (47.8%)	10 (55.6%)	421 (51.9%)	117 (49.8%)	126 (51.0%)	178 (54.1%)
**Marital status**								
Married	103 (46.2%)	56 (48.7%)	40 (44.4%)	7 (38.9%)	443 (54.6%)	178 (75.7%)	135 (54.7%)	130 (39.5%)
Single	43 (19.3%)	27 (23.5%)	14 (15.6%)	2 (11.1%)	110 (13.6%)	49 (20.9%)	48 (19.4%)	13 (4%)
Widowed	77 (34.5%)	32 (27.8%)	36 (40%)	9 (50.0%)	258 (31.8%)	8 (3.4%)	64 (25.9%)	186 (56.5%)
**Education level**								
Primary or no education	50 (22.5%)	21 (18.3%)	22 (24.4%)	7 (41.2%)	304 (37.7%)	30 (12.8%)	100 (40.5%)	174 (53.7%)
Secondary and above	172 (77.5%)	94 (81.7%)	68 (75.6%)	10 (58.8%)	502 (62.3%)	205 (87.2%)	147 (59.5%)	150 (46.3%)
**Employment status**								
Unemployed	148 (66.4%)	66 (57.4%)	65 (72.2%)	17 (94.4%)	646 (79.7%)	129 (54.9%)	198 (80.2%)	319 (97.0%)
Employed	75 (33.6%)	49 (42.6%)	25 (27.8%)	1 (5.6%)	165 (20.3%)	106 (45.1%)	49 (19.8%)	10 (3.0%)
**Wealth index tertiles**								
Low	41 (18.4%)	27 (23.5%)	11 (12.2%)	3 (16.7%)	112 (13.8%)	39 (16.6%)	33 (13.4%)	40 (12.2%)
Middle	140 (62.8%)	68 (59.1%)	60 (66.7%)	12 (66.7%)	514 (63.4%)	136 (57.9%)	164 (66.4%)	214 (65.0%)
High	42 (18.8%)	20 (17.4%)	19 (21.1%)	3 (16.7%)	185 (22.8%)	60 (25.5%)	50 (20.2%)	75 (22.8%)
**BMI categories**								
Underweight	22 (9.9%)	8 (7.0%)	10 (11.1%)	4 (22.2%)	47 (5.8%)	11 (4.7%)	17 (6.9%)	19 (5.8%)
Normal	100 (44.8%)	50 (43.5%)	46 (51.1%)	4 (22.2%)	343 (42.5%)	88 (37.6%)	98 (39.7%)	157 (48.0%)
Overweight	62 (27.8%)	38 (33.0%)	21 (23.3%)	3 (16.7%)	218 (27%)	69 (29.5%)	69 (27.9%)	80 (24.5%)
Obese	39 (17.5%)	19 (16.5%)	13 (14.4%)	7 (38.9%)	200 (24.8%)	63 (25.5%)	66 (28.2%)	71 (21.7%)
**Comorbid disease**								
Sensory impairment^a^	64 (29.5%)	23 (20.7%)	27 (30.7%)	14 (77.8%)	352 (44.1%)	47 (20.3%)	80 (33.2%)	225 (69.0%)
Joint conditions^b^	10 (4.5%)	3 (2.6%)	6 (6.7%)	1 (5.9%)	60 (7.4%)	5 (2.1%)	21 (8.5%)	34 (10.3%)
Cardiometabolic and renal conditions^c^	110 (49.3%)	41 (35.7%)	56 (62.2%)	13 (72.2%)	586 (72.3%)	124 (53.0%)	183 (74.1%)	279 (84.8%)
**Lifestyle factors**								
Former or current tobacco use	31 (13.9%)	17 (14.8%)	12 (13.3%)	2 (11.1%)	141 (17.4%)	38 (16.2%)	54 (21.9%)	49 (14.9%)
Consumes alcohol	48 (21.6%)	29 (25.2%)	18 (20.2%)	1 (5.6%)	128 (15.9%)	54 (23.0%)	51 (20.8%)	23 (7.0%)
**Self‐perception of health**								
EQ‐5D‐5L index value	87.0 ± 7.6	87.8 ± 5.1	87.1 ± 7.0	80.4 ± 16.6	84.6 ± 11.4	87.4 ± 6.8	86.3 ± 8.7	81.2 ± 14.6

*Note*: BMI was categorized as underweight (<18.5 kg m^2^), normal (18.5–24.9 kg m^2^), overweight (>25 and < 30 kg m^2^) and obese (≥30 kg m^2^) [35].

Abbreviations: BMI, body mass index; EQ‐5D‐5L, EuroQol‐5 Dimension 5 Level; SD, standard deviation.

^a^ Sensory impairment describes visual and hearing impairment.

^b^ Joint conditions include self‐reported diagnosis or taking medication for arthritis, rheumatoid arthritis and gout.

^c^ Cardiometabolic and renal conditions include undiagnosed hypertension and diabetes, self‐reported diagnosis or taking medication for hypertension, diabetes, hypercholesterolaemia, congestive cardiac failure, cardiovascular disease or atrial fibrillation.

A similar proportion of people living with HIV (52.1%) and without HIV (51.8%) were female. Cancer prevalence was 1.4% in the study population, and was more common in PLWH (2.4% vs. 1.2%; see Table S1). Prior tuberculosis was reported by 5.1%, being more common in those with HIV (16.2% vs. 2.5%) (Table S1).

### Prevalence of frailty criteria indicators and frailty in people with and without HIV

**FIGURE 1:**
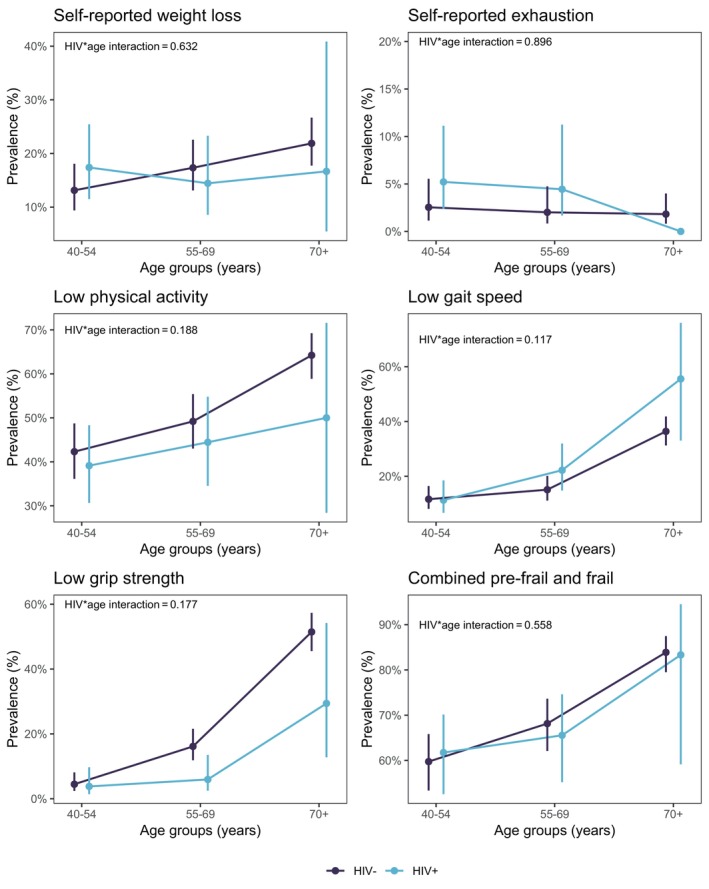
Prevalence (with 95% confidence Intervals) of frailty indicators and combined pre‐frail and frail groups (*p*‐values relate to the interaction between HIV and age). The confidence interval for self‐reported exhaustion in the 70+ age group in people living with HIV is not shown (it was 0–100%).

The prevalence of frailty was lower in people with HIV than in those without HIV (3.6% vs. 10.9% were frail, 61.4% vs. 61.1% were pre‐frail, and 35.0% vs. 27.9% were non‐frail). Figure 1 and Table S2 show the prevalence of the five frailty criteria, combined pre‐frailty and frailty in people with and without HIV, disaggregated by age group. Generally, confidence intervals of all prevalence estimates overlapped, denoting similarity between people with and without HIV in all age groups (Figure 1 and Table S2). Overall, in those aged 70+ years, 24.0% were frail and 60.2% were pre‐frail.

Table S3 shows factors associated with frailty: these included older age, being widowed, having primary or no formal education, wealth index, being unemployed, sensory loss, having been diagnosed with a cardiometabolic and/or renal conditions, or cancer. The association between HIV and frailty is shown in Table 3. Before adjustment, HIV was associated with lower odds of frailty (OR = 0.26, 95% CI: 0.12–0.55); however, adjusting for age attenuated this association to OR = 1.03 (95% CI: 0.44–2.40) (Table 3). After taking account of age, education level, marital status, BMI, wealth index and a cancer diagnosis, HIV was not associated with frailty [OR = 0.92 (0.64–1.30) for pre‐frailty vs. non‐frailty, and OR = 0.90 (0.38–2.13) for frailty vs. non‐frailty].

**TABLE 3:** The association between HIV and frailty before and after adjustment for age and further potential confounders.

	Pre‐frail vs. non‐frail	Frail vs. non‐frail
Model 1 (unadjusted)	0.79 (0.58–1.09)	0.26 (0.12–0.55)
Model 2 (Age‐adjusted)	1.04 (0.74–1.45)	1.03 (0.44–2.40)
Model 3 (Fully adjusted)	0.92 (0.64–1.30)	0.90 (0.38–2.13)

*Note*: Data are presented as odds ratios (95% confidence interval). Odds ratios are based on multinomial logistic regression models. Model 3 was adjusted for age, education level, marital status, body mass index, wealth index, and cancer diagnosis.

### Factors associated with frailty in people living with HIV

Age was most strongly associated with frailty; those aged 70+ had approximately seven‐fold higher odds of being pre‐frail or frail than those aged 55–69 and 40–54 years (Table 4). Each 5‐year period since diagnosis with HIV was associated with a doubling in the odds of frailty, after controlling for age and ART duration. While, after adjusting for age and time since diagnosis, each 5‐year period of ART use was associated with 61% lower odds of frailty. Few PLWH had a viral load ≥50 copies/mL (16.7%, *n* = 36), but those who did had 79% higher odds of frailty, albeit with a 95% CI inclusive of the null. After age adjustment, sociodemographic factors were associated with frailty; those with no education or primary‐level education had double the odds of frailty compared with secondary or tertiary‐level education. Further, compared with those in the low wealth tertile, those in the middle tertile had 51% lower odds of frailty.

**TABLE 4:** Factors associated with pre‐frailty and frailty among 223 older people living with HIV in Zimbabwe.

Variable	Frequency [*n* (%)] or mean ± SD	Associations with pre‐frailty and frailty		Associations with multimorbidity	
		Unadjusted odds ratio (95% CI)	Age‐adjusted odds ratio (95% CI)	Unadjusted odds ratio (95% CI)	Age‐adjusted odds ratio (95% CI)
***Sex***					
Women	116 (52%)	1		1	
Men	107 (48%)	0.76 (0.44–1.30)		0.91 (0.56–1.47)	
***Age group (years)***					
40–54	115 (51.6%)	1		1	
55–69	90 (40.4%)	1.24 (0.71–2.18)		**2.14 (1.28 (3.61)**	
≥70	18 (8.1%)	**6.85 (2.00–25.44)**		**7.63 (2.71–25.04)**	
***HIV diagnosis and treatment***					
Undiagnosed HIV	15 (6.7%)	2.21 (0.72–7.43)	2.20 (0.72–7.37)	1.10 (0.41–2.99)	1.02 (0.37–2.82)
Age at diagnosis (years) – per 5‐year increase	44.7 ± 10.4	**1.18 (1.03–1.37)**	0.99 (0.75–1.32)	1.38 (1.18–1.54)	1.06 (0.82–1.37)
Years since diagnosis – per 5‐year increase	9.8 ± 5.0	1.01 (0.77–1.34)	**2.03 (1.03–4.13**)^a^	0.97 (0.75–1.25)	1.16 (0.62–2.22)^a^
ART duration (years) – per 5‐year increase	9.4 ± 5.1	0.87 (0.66–1.14)	**0.39 (0.19–0.78)** ^b^	0.88 (0.68–1.14)	0.71 (0.37–1.33)^b^
***Viral load categories***					
<50 copies/mL	179 (83.3%)	1	1		
≥50 copies/mL	36 (16.7%)	1.55 (0.74–3.38)	1.79 (0.84–3.94)	0.94 (0.49–1.83)	1.14 (0.58–2.24)
***Sociodemographic characteristics***					
**Marital status**					
Married	103 (46.2%)	1	1		
Single	43 (19.3%)	0.56 (0.28–1.16)	0.73 (0.36–1.44)	1.18 (0.61–2.28)	1.42 (0.71–2.84)
Widowed	77 (34.5%)	1.22 (0.66–2.26)	1.34 (0.76–2.37)	1.54 (0.90–2.67)	1.31 (0.75–2.30)
**Education level**					
Secondary or tertiary	172 (77.5%)	1	1		
Primary or no education	50 (22.5%)	**2.24 (1.15–4.56)**	**2.02 (1.02–4.15)**	1.24 (0.69–2.24)	1.06 (0.58–1.95)
**Employment status**					
Employed	75 (33.6%)	1	1	1	1
Unemployed	148 (66.4%)	1.60 (0.91–2.80)	1.30 (0.73–2.33)	0.93 (0.56–1.55)	0.58 (0.33–1.00)
**Wealth index tertiles**					
Low	57 (25.6%)	1	1	1	1
Middle	96 (43.0%)	**0.49 (0.24–0.95)**	**0.49 (0.24–0.96)**	0.97 (0.51–1.83)	0.73 (0.37–1.43)
High	70 (31.4%)	0.66 (0.31–1.37)	0.54 (0.25–1.14)	0.89 (0.39–2.06)	0.85 (0.36–2.02)
**Lifestyle factors**					
Never used	192 (86.1%)	1	1	1	1
Current or former tobacco use	31 (13.9%)	1.36 (0.64–3.02)	1.37 (0.63–3.05)	0.85 (0.42–1.71)	0.84 (0.40–1.73)
Former or never drank	174 (78.4%)	1	1	1	1
Current alcohol intake	48 (21.6%)	0.79 (0.42–1.50)	0.88 (0.46–1.69)	0.86 (0.48–1.54)	1.02 (0.56–1.86)
**BMI categories**					
Underweight	22 (9.9%)	0.80 (0.31–2.08)	0.70 (0.27–1.84)	1.00 (0.43–2.32)	0.85 (0.35–2.05)
Normal	100 (44.8%)	1	1	1	1
Overweight	62 (27.8%)	1.02 (0.54–1.96)	1.08 (0.56–2.10)	0.89 (0.49–1.60)	0.97 (0.53–1.78)
Obese	39 (17.5%)	1.03 (0.49–2.20)	0.98 (0.46–2.12)	1.83 (0.92–3.70)	1.93 (0.95–4.01)

*Note*: BMI was categorized as underweight (<18.5 kg m^2^), normal (18.5–24.9 kg m^2^), overweight (>25 and <30 kg m^2^) and obese (≥30 kg m^2^) [28]. Bold odds ratios mean they do not include the null.

Abbreviations: ART, antiretroviral treatment; BMI, body mass index; SD, standard deviation; US$, US dollars.

^a^ Additionally adjusted for ART treatment period.

^b^ Additionally adjusted for years lived with HIV.

### The association between frailty and health‐related quality of life

**FIGURE 2:**
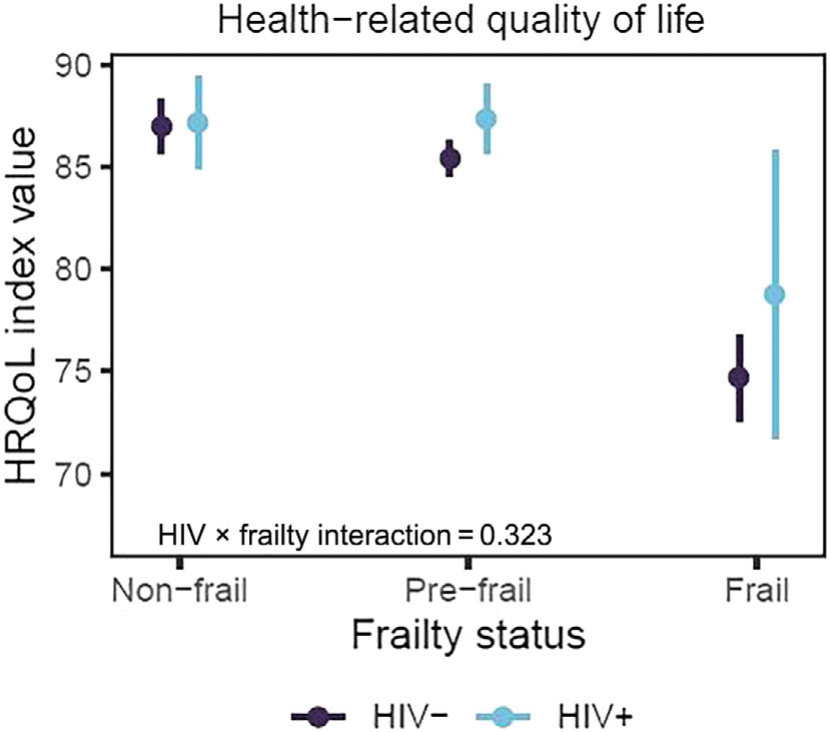
Health‐related quality of life (HRQoL) index values (with 95% confidence intervals) in non‐frail, pre‐frail and frail people with and without HIV (*p*‐value relates to the test for interaction between HIV and frailty status).

Figure 2 shows the HRQoL index values in non‐frail, pre‐frail and frail people with and without HIV. The HRQoL was lower in those who were frail than in those who were pre‐frail and non‐frail. There was no evidence of interaction between HIV status and frailty, meaning that the association between frailty and HRQoL was similar in people living with and without HIV.

## DISCUSSION

In this cross‐sectional study of middle‐aged and older adults in Zimbabwe, although 21.6% were living with HIV, few were older than 70 years. The prevalence rates of the five frailty criteria (weight loss, exhaustion, low physical activity, low gait speed, low grip strength) as well as frailty were similar in those with and without HIV. While HIV was not associated with frailty overall, a longer duration since diagnosis (independent of age and ART duration) was associated with frailty, whereas longer ART duration (independent of age and time since diagnosis) was associated with lower odds of frailty, and unsuppressed viral load (measured at a single time point) was only weakly associated with frailty. As expected, frailty was strongly associated with older age, as well as lower educational level. Furthermore, being frail was associated with lower HRQoL to a similar degree in both those with and without HIV.

The lack of association between HIV and frailty maybe explained by: (1) a survivor bias, in that people surviving with HIV to older age are a more resilient and robust population; and/or (2) the fact that untreated HIV is associated with frailty. Globally, it remains inconclusive as to whether HIV is associated with frailty. The pooled prevalence of frailty in people living with HIV aged ≥50 years from 26 studies was 11%, with a range of 3–29% [24]. This pooled prevalence is similar to the global pooled prevalence of 12% from 240 studies, the majority (57%) conducted in people aged ≥65 years [5]. Whether HIV accelerates ageing, which in turn predisposes to frailty, remains unclear, given the complex interplay between HIV, ART use and adherence, and multiple contributors of frailty [8]. In Africa, as in our current study, well‐controlled HIV was found not to be associated with frailty in middle‐aged adults in Senegal [10] and older adults in Tanzania [13] and South Africa [12]. The only two African studies to report an association between HIV and frailty included participants, half of whom were aged <40 years, potentially lowering the risk of survivor bias. In the first South African study, authors noted that they could not establish whether it was HIV infection or ART that was responsible for the association, but recommended early ART initiation to prevent frailty [11]. In the second study in Ethiopia, ART duration was not adjusted for in the multivariate model [16], despite a median of 1 year between diagnosis and ART initiation. Taken in this context, the lack of association between HIV and frailty in our study should be interpreted with caution, given potential survivor bias, but our findings are consistent with the hypothesis that frailty may be mitigated by early ART initiation [25]. However, while ART initiation and viral suppression are important, sustained immunosuppression could contribute to the increased the odds of frailty. Longitudinal studies are needed to elucidate this hypothesis.

Other factors associated with frailty in people living with HIV in this study included older age (as expected), low education level and household wealth. Associations between markers of socioeconomic deprivation and frailty are well established [26, 36], and have been reported in studies of people living with HIV in Africa [11, 12, 13, 16]. These factors should be considered during the design and deployment of interventions to mitigate frailty.

The HIV prevalence in this study is consistent with previous UNAIDS estimates [19]. Recent evidence suggests that 95‐95‐95 target achievement in Zimbabwe is heterogeneous and differs with geographical location, with poorer achievement mostly in the northern part of the country including Harare [18], where this study was conducted, suggesting the population sampled is representative of the underlying population. Our findings add evidence regarding the target's achievement in older people, who traditionally may not be considered at high HIV risk: more than 5% did not know they were living with HIV, and approximately 10% of those on ART were not virally suppressed. Late HIV diagnosis in older people is a global issue, with contributors including misperception of low risk by both older adults and healthcare workers, reducing testing opportunities, and some HIV symptoms being synonymous with ageing [35, 37]. Achievement of the 95‐95‐95 targets has important impacts on population dynamics and health. A modelling study in South Africa has shown that viral suppression through ART increases the life span and health span to similar levels as people living without HIV [38]. It could be that the effects of viral suppression through ART have not yet resulted in longer life span in this population, hence accounting for the low proportion of people living with HIV aged 70+ years in our study.

This study adds to the scant but emerging evidence on the associations between HIV and frailty in African populations. Interpretation of the findings should consider the following limitations. First, the cross‐sectional study design means causality and temporality cannot be established, and longitudinal studies are needed to confirm findings. Second, in determining frailty status, weight loss was self‐reported and not confirmed using weight measurements, which may have introduced recall bias. Additionally, self‐reported exhaustion may have been underestimated as we assessed it using a single binary question from the Shona Symptom Questionnaire [22] rather than two Likert questions used in the CES–D Depression Scale by Fried et al. [3]. Furthermore, there is no consensus or validity evidence to support the use of the question to assess exhaustion rather than contributing to screening of common mental disorders by the Shona Symptom Questionnaire [22] in this population. Physical activity may have been misreported due to use of the short form (rather than the long form) of the International Physical Activity Questionnaire [39], which has not been widely validated in Africa and does not have African language adaptations [40]. Also, some people living with HIV did not have written records for their date of diagnosis and ART initiation and hence they estimated it, which could be subject to recall bias. Finally, as previously highlighted, the lack of apparent association between HIV and frailty may be explained by survivor bias.

## CONCLUSIONS

Reduced survival to older age in those with HIV and good viral suppression may explain the lack of association between HIV and frailty. In PLWH, longer duration since HIV diagnosis (independent of age and ART duration) was associated with frailty, while longer use of ART (independent of age and time since diagnosis) was protective against frailty, as was HIV viral suppression, albeit weakly. Being older and having a lower education level and lower household wealth were associated with increased odds of being frail in people living with HIV. Our findings imply that early ART initiation may reduce frailty risk. Finally, interventions to prevent frailty in people living with HIV should particularly target those who are older, with low wealth and education levels.

## AUTHOR CONTRIBUTIONS

TM, TB, JC, AB and HW were responsible for data collection and management. AMM, RAF and CG were responsible for study conceptualization and design, and data interpretation. AMM analysed the data. AMM and CG were responsible for the first draft of the manuscript, which was critically reviewed and edited by TM, TB, JC, AB, HW, CG, YM, BC, KW and RAF. All authors read and approved the final version of the manuscript.

## FUNDING INFORMATION

This work was supported by the National Institute for Health Research (NIHR) [using the UK's Official Development Assistance (ODA) Funding]; CG, AMM and TM are funded via NIHR302394. The Fractures‐E [3] study was supported by the National Institute for Health Research (NIHR) (using the UK's ODA Funding) and Wellcome (217135/Z/19/Z) under the NIHR‐Wellcome Partnership for Global Health Research. The views expressed are those of the authors and not necessarily those of the NIHR, the Department of Health and Social Care or Wellcome. For the purpose of Open Access, the author has applied a CC‐BY public copyright licence to any Author Accepted Manuscript version arising from this submission.

## CONFLICT OF INTEREST STATEMENT

The authors declare there are no conflicts of interest.

## ETHICAL APPROVAL

Ethical approval was obtained from the following institutions: the Medical Research Council of Zimbabwe (MRCZ/A/2706); the Biomedical Research and Training Institute (AP161/2021); Sally Mugabe Central Hospital (HCHEC/250 121/06); the University of Zimbabwe College of Health Sciences and the Parirenyatwa group of hospitals (25/02/2021); and Harare City Health (27/01/2021). All participants provided written informed consent before data collection.

## Supporting information


**DATA S1.** Supporting Information.

## Data Availability

Data are available upon reasonable request. Researchers can access participant‐level data via data.bris, provided ethical approvals are in place.
