# Supplementary material for: Prevalence, factors and quality of life associated with frailty and pre‐frailty in middle‐aged and older adults living with HIV in Zimbabwe: A cross‐sectional study
Source: HIV Med. 2024 Sep 20;26(1):153–65. doi: 10.1111/hiv.13716 (PMC11725409; doi:10.1111/hiv.13716)
Supplement: Supplementary file 1 — DATA S1. Supporting Information. [file HIV-26-153-s001.docx]

**Supplementary File**

Table of Contents

[Figure 1 2](#_Toc163565467)

[Disease data collection and definitions 3](#_Toc163565468)

[Direct Acyclic Graph 4](#_Toc163565469)

[Supplementary Table 2: Prevalence of frailty indicators, prefrailty and frailty disaggregated by HIV status. 8](#_Toc163565470)

[Supplementary Table 3: Bivariate associations between frailty status and risk factors 9](#_Toc163565471)

[Wealth index generation 10](#_Toc163565472)

[STROBE Statement—Checklist of items that should be included in reports of *cross-sectional studies* 13](#_Toc163565473)

[References 16](#_Toc163565474)

# Figure 1


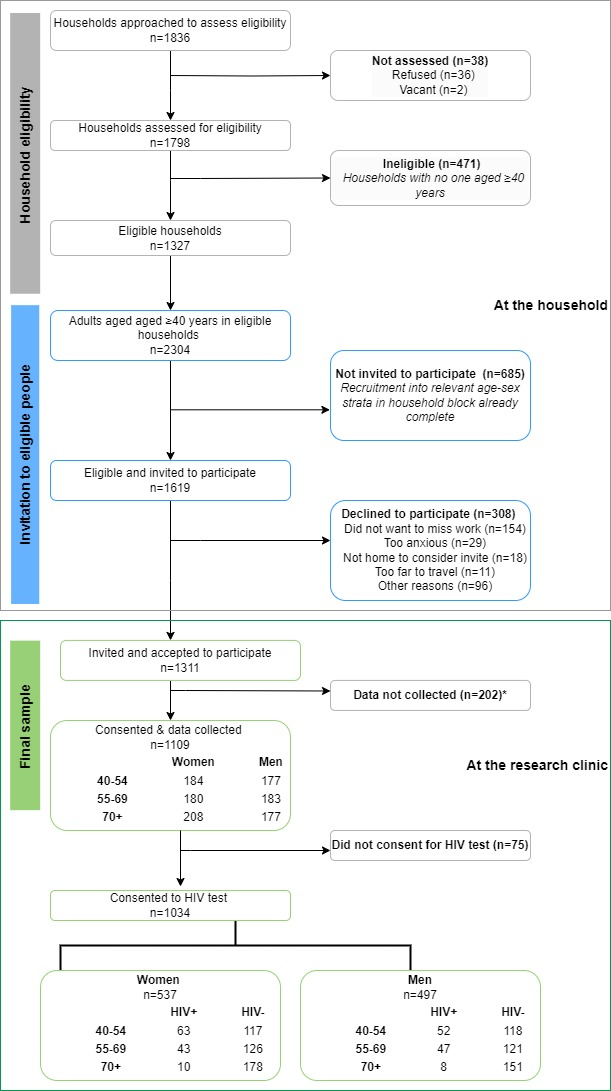


**Figure 1: Participant flow chart describing household-based identification and recruitment of study participants.**

** The study driver attempted to collect the participant from home on up to 3 occasions, each time the study team phoned to re-arrange the research clinic appointment. but the participant was not available.*

# Disease data collection and definitions

Hypertension was present if (i) previously diagnosed by a medical professional, (ii) taking antihypertensives or, (iii) the lowest reading was ≥140 mmHg for systolic blood pressure or ≥90 mmHg for diastolic blood pressure after at least three blood pressure measurements on the data collection day(1, 2).

Diabetes was defined (i) as a previous diagnosis by a medical professional, (ii) taking diabetes medications or, (iv) a random blood glucose (measured using a finger prick and glucometer) of >11mmol(3).

Mental health disorder was present if participants (i) scored ≥ 8 on the Shona Symptom Questionnaire(4), (ii) self-reported a diagnosis of a condition such as depression, anxiety or schizophrenia, or (iii) use of medicine(s) to treat a mental health disorder.

Pain was defined as experiencing pain other than normal forms of pain (e.g., minor headaches, sprains, and toothaches) in the last 48 hours, as defined using the Brief Pain Inventory(5).

Sensory loss was defined as having either visual or hearing impairment. Visual impairment was defined as a ‘fail’ for near vision (including a fail with glasses/contact lenses when applicable) or a moderate or severe distance vision impairment or blindness in either eye (including a fail with glasses/contact lenses when applicable). Hearing impairment was defined as self-reported hearing loss (Yes’ or ‘Sometimes’) or a hearWHO test (6) of <37.2 (this cut-off corresponded to the mean of the hearWHO test in those who self-reported hearing loss).

A history of falls was defined by self-report of at least one fall in the previous year. Presence of disability was based on the question ‘Do you consider yourself to be disabled?’. All other conditions were based on a self-reported diagnosis by a medical professional (‘Yes’, ‘No’, ‘Don’t know’), or the self-reported diagnostic indication for a medicine prescribed by a medical professional.

Polypharmacy was defined as use of five or more prescription medications taken as tablets(7) other than ART.

# Direct Acyclic Graph

This Direct Acyclic Graph was made using the DAGitty tool(8) and the content based on literature and brainstorming.


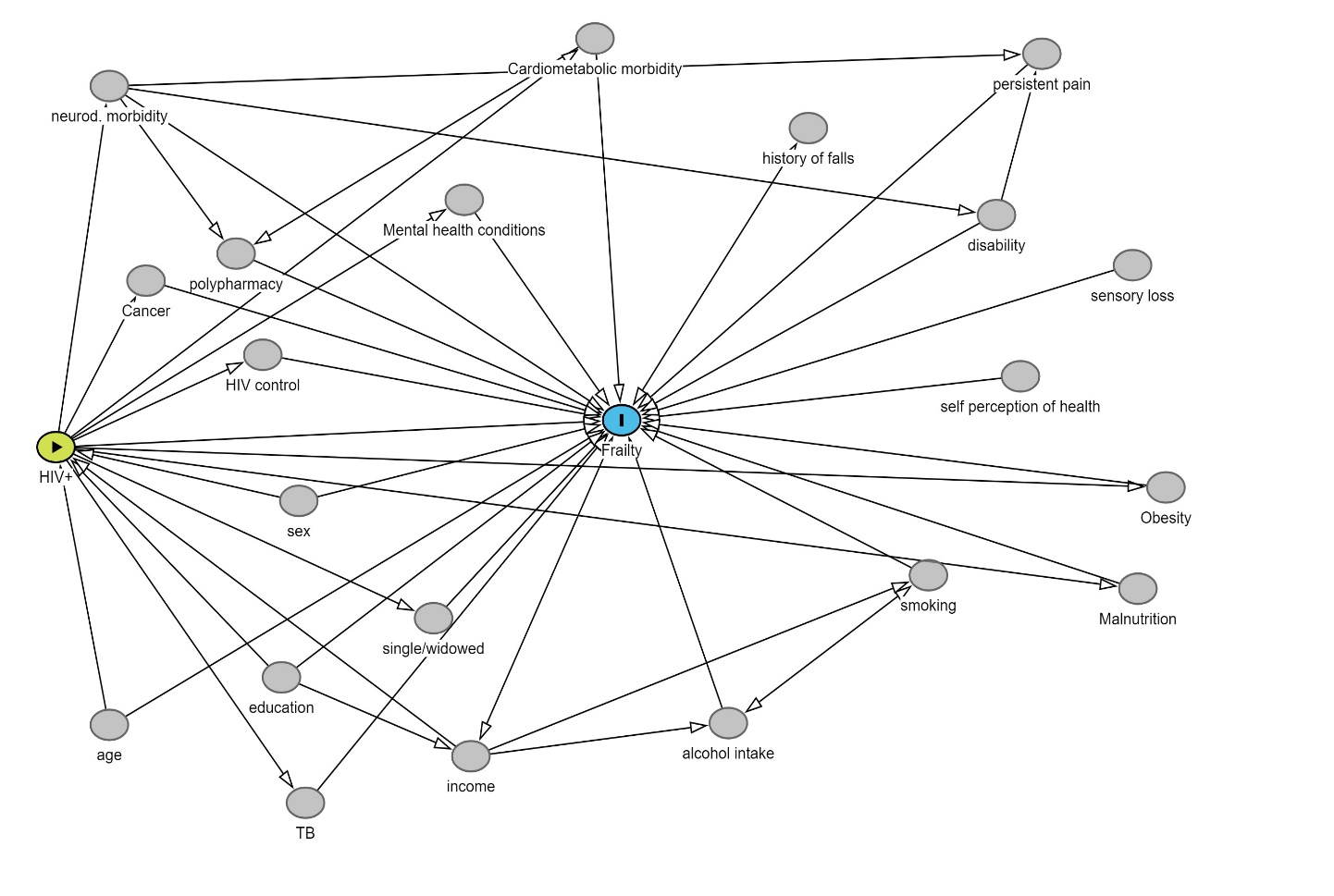


**Supplementary Figure 1: Direct Acyclic Graph showing risk factors for HIV and frailty.**

TB – Tuberculosis; neurod.morbidity – neurodegenerative morbidity

**Supplementary Table 1: Characteristics of all, people living with and without HIV disaggregated by three age strata.**

|  | **All**  **N=1109** | **HIV+, n=223 (21.6%)** | | | | **HIV-, n=811 (78.4%)** | | | | P value* |
| --- | --- | --- | --- | --- | --- | --- | --- | --- | --- | --- |
|  |  | **N=223** | **40-54**  n=115, 51.6% | **55-69**  n=90, 40.4% | **70+**  n=18, 8.1% | **N=811** | **40-54**  n=235, 29.0% | **55-69**  n=247, 30.5% | **70+**  n=329, 40.6% |  |
| **Sociodemographic characteristics** | | | | | | | | | | |
| **Female sex** | 572 (51.6%) | 116 (52.0%) | 63 (54.8%) | 43 (47.8%) | 10 (55.6%) | 421 (51.9%) | 117 (49.8%) | 126 (51.0%) | 178 (54.1%) | 0.816 |
| **Marital status** | | | | | | | | | | |
| Married | 579 (52.2%) | 103 (46.2%) | 56 (48.7%) | 40 (44.4%) | 7 (38.9%) | 443 (54.6%) | 178 (75.7%) | 135 (54.7%) | 130 (39.5%) | <0.001 |
| Single | 162 (14.6%) | 43 (19.3%) | 27 (23.5%) | 14 (15.6%) | 2 (11.1%) | 110 (13.6%) | 49 (20.9%) | 48 (19.4%) | 13 (4%) |  |
| Widowed | 368 (33.2%) | 77 (34.5%) | 32 (27.8%) | 36 (40%) | 9 (50.0%) | 258 (31.8%) | 8 (3.4%) | 64 (25.9%) | 186 (56.5%) |  |
| **Education level** | | | | | | | | | | |
| Primary or no education | 397 (36.0%) | 50 (22.5%) | 21 (18.3%) | 22 (24.4%) | 7 (41.2%) | 304 (37.7%) | 30 (12.8%) | 100 (40.5%) | 174 (53.7%) | 0.144 |
| Secondary and above | 705 (64.0%) | 172 (77.5%) | 94 (81.7%) | 68 (75.6%) | 10 (58.8%) | 502 (62.3%) | 205 (87.2%) | 147 (59.5%) | 150 (46.3%) |  |
| **Employment status** | | | | | | | | | | |
| Unemployed | 851 (76.7%) | 148 (66.4%) | 66 (57.4%) | 65 (72.2%) | 17 (94.4%) | 646 (79.7%) | 129 (54.9%) | 198 (80.2%) | 319 (97.0%) | 0.561 |
| Employed | 258 (23.3%) | 75 (33.6%) | 49 (42.6%) | 25 (27.8%) | 1 (5.6%) | 165 (20.3%) | 106 (45.1%) | 49 (19.8%) | 10 (3.0%) |  |
| **Wealth index tertiles** | | | | | | | | | | |
| Low | 152 (13.7%) | 57 (25.6%) | 34 (29.6%) | 18 (20%) | 5 (27.8%) | 87 (10.7%) | 41 (17.4%) | 24 (9.7%) | 22 (6.7%) | <0.001 |
| Middle | 489 (44.1%) | 96 (43%) | 58 (50.4%) | 33 (36.7%) | 5 (27.8%) | 361 (44.5%) | 110 (46.8%) | 119 (48.2%) | 132 (40.1%) |  |
| High | 468 (42.2%) | 70 (31.4%) | 23 (20%) | 39 (43.3%) | 8 (44.4%) | 363 (44.8%) | 84 (35.7%) | 104 (42.1%) | 175 (53.2%) |  |
| **BMI Categories** | | | | | | | | | | |
| Underweight | 75 (6.8%) | 22 (9.9%) | 8 (7.0%) | 10 (11.1%) | 4 (22.2%) | 47 (5.8%) | 11 (4.7%) | 17 (6.9%) | 19 (5.8%) | 0.013 |
| Normal | 477 (43.2%) | 100 (44.8%) | 50 (43.5%) | 46 (51.1%) | 4 (22.2%) | 343 (42.5%) | 88 (37.6%) | 98 (39.7%) | 157 (48.0%) |  |
| Overweight | 299 (27.1%) | 62 (27.8%) | 38 (33.0%) | 21 (23.3%) | 3 (16.7%) | 218 (27%) | 69 (29.5%) | 69 (27.9%) | 80 (24.5%) |  |
| Obese | 252 (22.8%) | 39 (17.5%) | 19 (16.5%) | 13 (14.4%) | 7 (38.9%) | 200 (24.8%) | 63 (25.5%) | 66 (28.2%) | 71 (21.7%) |  |
| **Comorbid disease** | | | | | | | | | | |
| Pain prevalence | 283 (26.0%) | 49 (22.0%) | 22 (19.1%) | 21 (23.3%) | 6 (33.3%) | 206 (25.4%) | 50 (21.3%) | 59 (23.9%) | 97 (29.5%) | 0.871 |
| History of falls | 219 (20.0%) | 38 (17.0%) | 19 (16.5%) | 13 (14.4%) | 6 (33.3%) | 173 (21.3%) | 41 (17.4%) | 47 (19.0%) | 85 (25.8%) | 0.689 |
| Disability | 38 (3.4%) | 2 (0.9%) | 1 (0.9%) | 0 (0%) | 1 (0.9%) | 33 (4.1%) | 7 (3.0%) | 6 (2.4%) | 20 (6.1%) | 0.151 |
| **Sensory impairment – either** | 461 (42.4%) | 64 (29.5%) | 23 (20.7%) | 27 (30.7%) | 14 (77.8%) | 352 (44.1%) | 47 (20.3%) | 80 (33.2%) | 225 (69.0%) | 0.968 |
| *Visual impairment* | 358 (33%) | 53 (24%) | 19 (16.5%) | 24 (27.3%) | 10 (55.6%) | 273 (34.3%) | 32 (13.7%) | 63 (25.7%) | 178 (56.0%) | 0.615 |
| *Hearing impairment* | 263 (24.2%) | 23 (10.6%) | 6 (5.4%) | 10 (11.4%) | 7 (38.9%) | 211 (26.4%) | 22 (9.4%) | 34 (14.2%) | 155 (47.7%) | 0.161 |
| Mental health conditions | 8 (0.7%) | 1 (0.4%) | 1 (0.9%) | 0 (0%) | 0 (0%) | 7 (0.9%) | 2 (0.9%) | 4 (1.6%) | 1 (0.3%) | 0.619 |
| Prior Tuberculosis | 57 (5.1%) | 36 (16.1%) | 22 (19.1%) | 14 (15.6%) | 0 (0%) | 20 (2.5%) | 2 (0.9%) | 10 (4.0%) | 8 (2.4%) | <0.001 |
| Respiratory conditions | 29 (2.6%) | 6 (2.7%) | 3 (2.6%) | 3 (3.3%) | 0 (0%) | 21 (2.6%) | 4 (1.7%) | 7 (2.8%) | 10 (3.1%) | 0.998 |
| Cancer | 15 (1.4%) | 5 (2.3%) | 3 (2.6%) | 1 (1.1%) | 1 (5.9%) | 10 (1.2%) | 1 (0.4%) | 2 (0.8%) | 7 (2.1%) | 0.141 |
| Neurodegenerative conditions | 47 (4.2%) | 6 (2.7%) | 3 (2.6%) | 1 (1.1%) | 2 (11.8%) | 40 (4.9%) | 11 (4.7%) | 13 (5.3%) | 16 (4.9%) | 0.280 |
| Joint conditions | 80 (7.2%) | 10 (4.5%) | 3 (2.6%) | 6 (6.7%) | 1 (5.9%) | 60 (7.4%) | 5 (2.1%) | 21 (8.5%) | 34 (10.3%) | 0.710 |
| Cardiometabolic and renal conditions | 749 (67.6%) | 110 (49.3%) | 41 (35.7%) | 56 (62.2%) | 13 (72.2%) | 586 (72.3%) | 124 (53.0%) | 183 (74.1%) | 279 (84.8%) | <0.001 |
| Polypharmacy | 7 (1.4%) | 0 (0%) | 0 (0%) | 0 (0%) | 0 (0%) | 7 (1.9%) | 1 (1.8%) | 1 (1.0%) | 5 (2.4%) | 0.478 |
| **Lifestyle factors** | | | | | | | | | | |
| Former or current tobacco use | 190 (17.1%) | 31 (13.9%) | 17 (14.8%) | 12 (13.3%) | 2 (11.1%) | 141 (17.4%) | 38 (16.2%) | 54 (21.9%) | 49 (14.9%) | 0.152 |
| **Current alcohol intake** | 194 (17.6%) | 48 (21.6%) | 29 (25.2%) | 18 (20.2%) | 1 (5.6%) | 128 (15.9%) | 54 (23.0%) | 51 (20.8%) | 23 (7.0%) | 0.908 |
| **Self-perception of health** | | | | | | | | | | |
| EQ-5D-5L index value | 84.9±11.2 | 87.0±7.6 | 87.8±5.1 | 87.1±7.0 | 80.4±16.6 | 84.6±11.4 | 87.4±6.8 | 86.3±8.7 | 81.2±14.6 | 0.002 |
| *P values based on Mantel-Haenszel test for categorical variables or Analysis of Variance (ANOVA) for continuous variables adjusted for age-group.  BMI – body mass index; EQ-5D-5L – EuroQol-5 Dimension 5 Level; SD – standard deviation; USD – US Dollars | | | | | | | | | | |

# Supplementary Table 2: Prevalence of frailty indicators, prefrailty and frailty disaggregated by HIV status.

|  | **HIV+, n=223 (21.6%)** | | | **HIV-, n=811 (78.4%)** | | | **HIV status* age interaction P value** |
| --- | --- | --- | --- | --- | --- | --- | --- |
|  | **40-54**  n=115, 51.6% | **55-69**  n=90, 40.4% | **70+**  n=18, 8.1% | **40-54**  n=235, 29.0% | **55-69**  n=247, 30.5% | **70+**  n=329, 40.6% |  |
| **Frailty indicators - % (95% CI)** | | | | | | | |
| Weight loss | 17.4%  (11.5, 24.4) | 14.4%  (8.6, 23.3) | 16.7%  (5.5, 40.9) | 13.2 %  (9.4, 18.2) | 17.4 %  (13.2, 22.7) | 21.9%  (17.7, 26.7) | 0.632 |
| Exhaustion | 5%  (2, 11.1) | 4%  (2, 11.3) | 0%  (0, 100) | 2.6%  (1.2, 5.6) | 2.0%  (0.8, 4.8) | 1.8%  (0.8, 4.0) | 0.896 |
| Low physical activity | 39.1%  (30.6, 48.3) | 44.4%  (34.5, 54.8) | 50.0% (28.4, 71.6) | 41.2%  (35.1, 47.6) | 49.4%  (43.2, 55.6) | 64.5%  (59.2, 69.5) | 0.188 |
| Low gait speed | 11.3 %  (6.7, 18.5) | 22.2%  (14.8, 32.0) | 55.6% (33.0, 76.0) | 11.7%  (8.1, 16.5) | 15.2%  (11.2, 20.2) | 36.5%  (31.4, 42.0) | 0.117 |
| Low grip strength | 3.7%  (1.4, 9.4) | 5.3%  (2.2, 12.3) | 30.6% (13.2, 56.2) | 4.1%  (2.2, 7.7) | 16.0%  (11.7, 21.5) | 51.2%  (45.2, 57.1%) | 0.177 |
| **Frailty status- % (95% CI)** | | | | | | | |
| Combined pre-frail and frail | 61.7%  (52.5, 70.2) | 65.6%  (55.2, 74.6) | 83.3% (59.1, 94.5) | 60.0%  (53.6, 66.1) | 68.4%  (62.4, 73.9) | 84.1%  (79.8, 87.7) | 0.558 |

# Supplementary Table 3: Bivariate associations between frailty status and risk factors

|  | **All**  **N=1109** | **Non-frail**  **n=320** | **Pre-frail**  **n=678** | **Frail**  **n=110** | **P value** |
| --- | --- | --- | --- | --- | --- |
| **Sex** | | | | | |
| Women | 572 (51.6%) | 158 (27.7%) | 353 (61.8%) | 60(10.5%) | 0.545 |
| Men | 537 (48.4%) | 162 (30.2%) | 325 (60.5%) | 50 (9.3%) |  |
| **Age** | 62.5±14.1 | 57.1±11.7 | 62.5±13.8 | 77.9±10.4 | <0.001 |
| **Marital status** | | | | | |
| Married | 579 (52.2%) | 178 (30.7%) | 362 (62.5%) | 39 (6.7%) | <0.001 |
| Single | 162 (14.6%) | 62 (38.3%) | 88 (54.3%) | 12 (7.4%) |  |
| Widowed | 368 (33.2%) | 80 (21.8%) | 228 (62.1%) | 59 (16.1%) |  |
| **Education level** | | | | | |
| Primary or no education | 397 (36.0%) | 52 (13.1%) | 272 (68.7%) | 72 (18.2%) | <0.001 |
| Secondary and above | 705 (64.0%) | 267 (37.9%) | 402 (57.0%) | 36 (5.1%) |  |
| **Employment status** | | | | | |
| Unemployed | 851 (76.7%) | 194 (22.8%) | 549 (64.6%) | 107 (12.6%) | <0.001 |
| Employed | 258 (23.2%) | 126 (48.8%) | 129 (50.0%) | 3 (1.2%) |  |
| **Wealth index tertiles** | | | | | |
| Low | 152 (13.7%) | 24 (7.5%) | 114 (16.8%) | 14 (12.7%) | 0.002 |
| Middle | 489 (44.1%) | 151(47.2%) | 293 (43.2%) | 45 (40.9%) |  |
| High | 468 (42.2%) | 145 (45.3%) | 272 (40.1%) | 51 (46.4%) |  |
| **Body composition** | | | | | |
| Underweight | 75 (6.8%) | 22 (29.3%) | 40 (53.3%) | 13 (17.3%) | 0.015 |
| Normal | 477 (43.2%) | 138 (28.9%) | 279 (58.5%) | 60 (12.6%) |  |
| Overweight | 299 (27.1%) | 89 (29.8%) | 192 (64.2%) | 18 (6.0%) |  |
| Obese | 252 (22.8%) | 71 (28.2%) | 162 (64.3%) | 19 (7.5%) |  |
| **Comorbid disease** | | | | | |
| **Pain presence** | | | | | |
| Yes | 283 (25.5%) | 98 (34.6% | 152 (53.7%) | 33 (11.7%) | 0.011 |
| No | 826 (74.5% | 222 (26.9%) | 526 (63.8%) | 77 (9.3%) |  |
| **Ever fallen** | | | | | |
| Yes | 219 (19.7%) | 59 (26.9%) | 133 (60.7%) | 27 (12.3%) | 0.377 |
| No | 890 (80.2%) | 261 (29.4%) | 545 (61.3%) | 83 (9.3%) |  |
| **Self-reported disability** | | | | | |
| Yes | 38 (3.4%) | 3 (7.9%) | 15 (39.5%) | 20 (52.6%) | <0.001 |
| No | 1071 (96.6%) | 333 (31.1%) | 653 (61.0%) | 85 (7.9%) |  |
| **Sensory impairment – either** | | | | | |
| Yes | 461 (42.4%) | 99 (21.5%) | 278 (60.3%) | 84 (18.2%) | <0.001 |
| No | 625 (57.6%) | 216 (34.6%) | 385 (61.6%) | 24 (3.8%) |  |
| *Visually impaired* | | | | | |
| Yes | 358 (32.9%) | 72 (20.1%) | 218 (60.9%) | 68 (19.0%) | <0.001 |
| No | 730 (67.1%) | 245 (33.6%) | 451 (61.8%) | 34 (4.7%) |  |
| *Hearing impairment* | | | | | |
| Yes | 263 (24.2%) | 44 (16.7%) | 159 (60.5%) | 60 (22.8%) | <0.001 |
| No | 822 (75.8%) | 272 (33.1%) | 501 (60.9%) | 49 (6.0%) |  |
| **Mental health conditions** | | | | | |
| Yes | 8 (0.7%) | 2 (25.0%) | 5 (62.5%) | 1 (12.5%) | 0.877 |
| No | 1101 (99.3%) | 334 (30.3%) | 663 (60.2%) | 104 (9.4%) |  |
| **Prior Tuberculosis** | | | | | |
| Yes | 57 (5.2%) | (22.8%) | 39 (68.4%) | 5 (8.8%) | 0.575 |
| No | 1048 (94.8%) | 306 (29.2%) | 637 (60.8%) | 104 (9.9%) |  |
| **Asthma** | | | | | |
| Yes | 29 (2.6%) | 12 (41.4%) | 14 (48.3%) | 3 (10.3%) | 0.352 |
| No | 1077 (97.4%) | 324 (30.1%) | 652 (60.5%) | 101 (9.4%) |  |
| **Cancer** | | | | | |
| Yes | 15 (1.4%) | 0 (0%) | 11 (78.6%) | 3 (21.4%) | 0.011 |
| No | 1090 (98.6%) | 320 (29.4%) | 664 (60.9%) | 101 (9.3%) |  |
| **Neurodegenerative conditions** | | | | | |
| Yes | 47 (4.2%) | 5 (10.6%) | 24 (51.1%) | 18 (38.3%) | <0.001 |
| No | 1060 (95.8%) | 315 (29.7%) | 652 (61.6%) | 106 (9.7%) |  |
| **Joint conditions** | | | | | |
| Yes | 80 (7.2%) | 16 (20%) | 54 (67.5%) | 10 (12.5%) | 0.173 |
| No | 1028 (92.8%) | 304 (29.6%) | 623 (60.7%) | 100 (9.7%) |  |
| **Cardiometabolic and renal conditions** | | | | | |
| Yes | 749 (67.6%) | 193 (25.8%) | 467 (62.4%) | 88 (11.8%) | <0.001 |
| No | 359 (32.4%) | 127 (35.4%) | 210 (58.5%) | 22 (6.1%) |  |
| **Polypharmacy** | | | | | |
| Yes | 7 (1.4%) | 2 (28.6%) | 5 (71.4%) | 0 (0%) | 0.739 |
| No | 504 (98.6%) | 109 (21.7%) | 327 (65%) | 67 (13.3%) |  |
| **Lifestyle factors** | | | | | |
| **Tobacco use** | | | | | |
| Never used | 919 (82.9%) | 265 (28.9%) | 562 (61.2%) | 91 (9.9%) | 0.476 |
| Former user | 39 (3.5%) | 9 (23.1%) | 23 (59%) | 7 (17.9%) |  |
| Current user | 151 (13.6%) | 46 (30.5%) | 93 (61.6%) | 12 (7.9%) |  |
| **Current alcohol intake** | | | | | |
| Yes | 194 (17.6%) | 76 (39.2%) | 114 (58.8%) | 4 (2.1%) | <0.001 |
| No | 910 (82.4%) | 244 (26.8%) | 560 (61.6%) | 105 (11.6%) |  |
| **Self-perception of health** | | | | | |
| EQ-5D-5L index value | 84.9±11.2 | 86.9±5.8 | 85.5±10.1 | 75.3±20.1 | <0.001 |

# Wealth index generation

The wealth index was generated using Principal Component Analysis combining house ownership, housing characteristics (roof and wall material) and ownership of household assets (e.g. television, refrigerator, car). The index generation was done in R statistical program (version 4.3.1)(9) using ‘psych’ package(10). Table 4 shows the variables used in developing the wealth index. Two analysis were done: one using all 18 variables and one excluding three variables that had a Kaiser-Meyer-Olkin index of <0.5(11). Table 5 shows the specifications of the formulae and results when generating the index in the two analyses. In the second analysis, six factors were extracted which corresponded to Eigenvalues of ≥0.93 and communality mean of 0.6 (11). Both orthogonal and oblique component rotations were attempted for both analyses. However, this resulted in skewed wealth index distribution hence dropped and none of the rotations were used. Results from the second analysis were used in estimating wealth and tertiles of the index were used. Table 6 shows percentage of house ownership, housing characteristics and asset ownership in the three wealth tertiles based on the second index generation analysis. Generally, there was an increase in most asset ownership with wealth validating the use of the index in quantifying wealth.

**Supplementary Table 4: House ownership, housing characteristics and asset ownership variables used to generate the wealth index.**

| **Item** | **Definition as binary variable** | **Analysis 1** | **Analysis 2** |
| --- | --- | --- | --- |
| 1. Household ownership | Used as binary: owned dwelling or renting main dwelling as 1 and rent part of dwelling or use dwelling without paying rent as 0 | Included | Included |
| 1. Main material of the exterior walls of dwelling | Higher quality wall material (cement) as 1 and low-quality material (wood, mud, other) as 0 | Included | Included |
| 1. Main material of the roof of dwelling | Higher quality roof material (tile, asbestos, corrugate iron) as 1 and low-quality material (wood, other) as 0 | Included | Excluded as Kaiser-Meyer-Olkin index is <0.5 |
| 1. Electricity | Yes (1), No (0) | Included | Included |
| 1. Refrigerator | Yes (1), No (0) | Included | Included |
| 1. Television | Yes (1), No (0) | Included | Included |
| 1. Working car/truck | Yes (1), No (0) | Included | Included |
| 1. Tap in house | Yes (1), No (0) | Included | Included |
| 1. Private water/borehole (running water) | Yes (1), No (0) | Included | Included |
| 1. Flush toilet | Yes (1), No (0) | Included | Included |
| 1. Pit latrine | Yes (1), No (0) | Included | Included |
| 1. Solar energy | Yes (1), No (0) | Included | Included |
| 1. Power generator | Yes (1), No (0) | Included | Included |
| 1. Satellite dish/decoder | Yes (1), No (0) | Included | Included |
| 1. Computer | Yes (1), No (0) | Included | Included |
| 1. Tiled floors | Yes (1), No (0) | Included | Included |
| 1. Plough | Yes (1), No (0) | Included | Excluded as Kaiser-Meyer-Olkin index is <0.5 |
| 1. Axe or hoe | Yes (1), No (0) | Included | Excluded as Kaiser-Meyer-Olkin index is <0.5 |

**Supplementary Table 5:** **Specifications of the formulae and results in generating wealth index index**

| **Analysis** | **Items used** | **Factors extracted** | **Results: Quintiles** | **Results: Tertiles** |
| --- | --- | --- | --- | --- |
| Analysis 1 | 18 | 18 (Eigenvalues range 2.5 to 0.5, communality mean=1) | Lower: 5.2%  Lower Middle: 16.9%  Middle: 45.4%  Upper Middle: 27.1%  Upper: 5.4% | Low: 15.0%  Middle: 62.6%  High: 22.4% |
| Analysis 2 | 15 | 6 (Eigenvalues 2.7 to 0.9, communality mean=0.6) | Lower: 5.3%  Lower Middle: 12.8%  Middle: 24.3%  Upper Middle: 47.6%  Upper: 10.0% | Low: 13.7%  Middle: 44.1%  High: 42.2% |
| Both orthogonal and oblique component rotations were attempted for both analyses. However, this resulted in skewed wealth index distribution hence was dropped and none of the rotations were used. | | | | |

**Supplementary Table 6: Percentage of house ownership, house characteristics, asset ownership in the generated wealth tertiles**

|  | **Wealth tertiles** | | |
| --- | --- | --- | --- |
|  | **Low** | **Middle** | **High** |
| Household ownership§ | 6.6 | 73.6 | 99.8 |
| Better quality wall§ | 100 | 99.2 | 99.6 |
| Better quality roof† | 100 | 99.8 | 99.8 |
| Electricity§ | 83.6 | 93.9 | 99.1 |
| Refrigerator | 26.3 | 69.9 | 98.1 |
| Television | 38.8 | 84.7 | 98.4 |
| Working car/truck | 3.3 | 12.3 | 29.5 |
| Tap in house§ | 33.6 | 50.9 | 75.9 |
| Private water/borehole (running water) § | 3.3 | 3.3 | 6.2 |
| Flush toilet§ | 44.1 | 65.0 | 87.8 |
| Pit latrine§ | 20.4 | 8.0 | 3.6 |
| Solar energy§ | 5.3 | 7.6 | 8.8 |
| Power generator | 0.7 | 1.4 | 7.1 |
| Satellite dish/decoder | 2.0 | 10.8 | 53.0 |
| Computer | 2.0 | 5.3 | 14.5 |
| Tiled floors | 14.5 | 20.2 | 40.0 |
| Plough† | 17.8 | 11.2 | 10.3 |
| Axe or hoe† | 44.1 | 64.6 | 69.4 |
| **Household income** | | | |
| No income | 17.7 | 51.7 | 30.5 |
| ≤100 USD | 15.7 | 44.9 | 39.3 |
| >100-200 USD | 9.0 | 40.1 | 50.9 |
| >200 USD | 4.5 | 31.3 | 64.3 |
| †Excluded from second analysis as Kaiser-Meyer-Olkin index is <0.5  §The Kaiser-Meyer-Olkin index for these variables was average i.e. >0.5 and <0.7 | | | |

# STROBE Statement—Checklist of items that should be included in reports of *cross-sectional studies*

|  | **Item No** | **Recommendation** | **Page reported** |
| --- | --- | --- | --- |
| **Title and abstract** | 1 | (*a*) Indicate the study’s design with a commonly used term in the title or the abstract | Title and page 1 |
|  |  | (*b*) Provide in the abstract an informative and balanced summary of what was done and what was found | 1 |
| **Introduction** | | |  |
| Background/rationale | 2 | Explain the scientific background and rationale for the investigation being reported | 3-4 |
| Objectives | 3 | State specific objectives, including any prespecified hypotheses | 4 |
| **Methods** | | |  |
| Study design | 4 | Present key elements of study design early in the paper | 1 and 4 |
| Setting | 5 | Describe the setting, locations, and relevant dates, including periods of recruitment, exposure, follow-up, and data collection |  |
| Participants | 6 | (*a*) Give the eligibility criteria, and the sources and methods of selection of participants | 4 |
| Variables | 7 | Clearly define all outcomes, exposures, predictors, potential confounders, and effect modifiers. Give diagnostic criteria, if applicable | 5-6 |
| Data sources/ measurement | 8* | For each variable of interest, give sources of data and details of methods of assessment (measurement). Describe comparability of assessment methods if there is more than one group | 5-6 and Supplementary material |
| Bias | 9 | Describe any efforts to address potential sources of bias | 9 |
| Study size | 10 | Explain how the study size was arrived at | 4 |
| Quantitative variables | 11 | Explain how quantitative variables were handled in the analyses. If applicable, describe which groupings were chosen and why | 9 |
| Statistical methods | 12 | (*a*) Describe all statistical methods, including those used to control for confounding | 9 |
|  |  | (*b*) Describe any methods used to examine subgroups and interactions | 9 |
|  |  | (*c*) Explain how missing data were addressed | NA |
|  |  | (*d*) If applicable, describe analytical methods taking account of sampling strategy | NA |
|  |  | (*e*) Describe any sensitivity analyses | NA |
| **Results** | | |  |
| Participants | 13* | (a) Report numbers of individuals at each stage of study—eg numbers potentially eligible, examined for eligibility, confirmed eligible, included in the study, completing follow-up, and analysed | 10 and Figure 1 |
|  |  | (b) Give reasons for non-participation at each stage | Figure 1 |
|  |  | (c) Consider use of a flow diagram |  |
| Descriptive data | 14* | (a) Give characteristics of study participants (eg demographic, clinical, social) and information on exposures and potential confounders | 10 and Table 1 |
|  |  | (b) Indicate number of participants with missing data for each variable of interest | 10 |
| Outcome data | 15* | Report numbers of outcome events or summary measures | Table 1 |
| Main results | 16 | (*a*) Give unadjusted estimates and, if applicable, confounder-adjusted estimates and their precision (eg, 95% confidence interval). Make clear which confounders were adjusted for and why they were included | Table 3, page 9 |
|  |  | (*b*) Report category boundaries when continuous variables were categorized | 6, Supplementary material, Table footnotes |
|  |  | (*c*) If relevant, consider translating estimates of relative risk into absolute risk for a meaningful time period | NA |
| Other analyses | 17 | Report other analyses done—eg analyses of subgroups and interactions, and sensitivity analyses | Supplementary material |
| **Discussion** | | |  |
| Key results | 18 | Summarise key results with reference to study objectives | 16 |
| Limitations | 19 | Discuss limitations of the study, taking into account sources of potential bias or imprecision. Discuss both direction and magnitude of any potential bias | 17 |
| Interpretation | 20 | Give a cautious overall interpretation of results considering objectives, limitations, multiplicity of analyses, results from similar studies, and other relevant evidence | 16-17 |
| Generalisability | 21 | Discuss the generalisability (external validity) of the study results | 16-17 |
| **Other information** | | |  |
| Funding | 22 | Give the source of funding and the role of the funders for the present study and, if applicable, for the original study on which the present article is based | Title page |

*Give information separately for exposed and unexposed groups.

**Note:** An Explanation and Elaboration article discusses each checklist item and gives methodological background and published examples of transparent reporting. The STROBE checklist is best used in conjunction with this article (freely available on the Web sites of PLoS Medicine at http://www.plosmedicine.org/, Annals of Internal Medicine at http://www.annals.org/, and Epidemiology at http://www.epidem.com/). Information on the STROBE Initiative is available at www.strobe-statement.org.

# References

1. Chalmers J, MacMahon S, Mancia G, Whitworth J, Beilin L, Hansson L, et al. 1999 World Health Organization-International Society of Hypertension Guidelines for the management of hypertension. Guidelines sub-committee of the World Health Organization. Clin Exp Hypertens. 1999;21(5-6):1009-60.

2. Muntner P, Shimbo D, Carey RM, Charleston JB, Gaillard T, Misra S, et al. Measurement of Blood Pressure in Humans: A Scientific Statement From the American Heart Association. Hypertension. 2019;73(5):e35-e66.

3. Organization WH. Definition and diagnosis of diabetes mellitus and intermediate hyperglycaemia: report of a WHO/IDF consultation. 2006.

4. Patel V, Simunyu E, Gwanzura F, Lewis G, Mann A. The Shona Symptom Questionnaire: the development of an indigenous measure of common mental disorders in Harare. Acta Psychiatr Scand. 1997;95(6):469-75.

5. Cleeland CS, Ryan K. The brief pain inventory. Pain Research Group. 1991;20(20):143-7.

6. WHO. Deafness and hearing loss: Hearing checks and the hearWHO app 2022 [Available from: <https://www.who.int/news-room/questions-and-answers/item/deafness-and-hearing-loss-hearing-checks-and-the-hearwho-app#:~:text=The%20hearWHO%20app%20provides%20a,tested%20by%20a%20trained%20professional>.

7. Masnoon N, Shakib S, Kalisch-Ellett L, Caughey GE. What is polypharmacy? A systematic review of definitions. BMC Geriatr. 2017;17(1):230.

8. Textor J, Hardt J, Knüppel S. DAGitty: A Graphical Tool for Analyzing Causal Diagrams. Epidemiology. 2011;22(5).

9. R Developement Core Team. R: A language and environment for statistical computing. (No Title). 2010.

10. Revelle W, Revelle MW. Package ‘psych’. The comprehensive R archive network. 2015;337(338).

11. Bikos LH. ReCentering Psych Stats: Psychometrics2022.
